# Posing the rationale for synthetic lipoxin mimetics as an adjuvant treatment to gold standard atherosclerosis therapies

**DOI:** 10.3389/fphar.2023.1125858

**Published:** 2023-02-14

**Authors:** Braden Millar, Monica de Gaetano

**Affiliations:** Diabetes Complications Research Centre, Conway Institute & School of Biomolecular and Biomedical Science, University College Dublin, Dublin, Ireland

**Keywords:** cardiovascular disease, efferocytosis, resolution pharmacology, synthetic lipoxin mimetics, FPR2, lipid-lowering drugs, glucose-lowering drugs

## Abstract

Atherosclerosis is a progressive, multifactorial inflammatory, and dyslipidaemic disease, responsible for the majority of cardiovascular diseases globally. The chronic inflammation is the main driver of the initiation and progression of such disease, as a result of an imbalanced lipid metabolism and an ineffective immune response to attenuate the inflammatory component. The importance of inflammation resolution is being increasingly recognised in atherosclerosis and cardiovascular disease. It has a complex mechanism consisting of multiple stages, including restoring an effective removal of apoptotic bodies (efferocytosis) and their degradation (effero-metabolism), a macrophage phenotype switching towards resolving phenotypes, and the promotion of tissue healing and regeneration. The low-grade inflammation associated with atherosclerosis development is a driving force in disease exacerbation, and hence inflammation resolution is a key area of research. In this review, we explore the complex disease pathogenesis and its many contributing factors to gain a greater understanding of the disease and identify the current and potential therapeutic targets. First-line treatments and their efficacy will also be discussed in detail, to highlight the emerging field of resolution pharmacology. Despite the great efforts made by current gold-standard treatments, such as lipid-lowering and glucose-lowering drugs, they remain ineffective at tackling residual inflammatory risk and residual cholesterol risk. Resolution pharmacology represents a new era of atherosclerosis therapy, as endogenous ligands associated with inflammation resolution are exploited for their pharmacological benefits in a more potent and longer-acting manner. Novel FPR2-agonists, such as synthetic lipoxin analogues, provide an exciting new approach to enhance the pro-resolving response of the immune system and subsequently end the pro-inflammatory response to allow for an anti-inflammatory and pro-resolving environment for tissue healing, regeneration, and return to homeostasis.

## Atherosclerosis: The ‘Silent Killer’ of the 21st century

Cardiovascular disease (CVD) is a huge umbrella term for several conditions, disorders and diseases of the vascular system and the heart [Including Coronary Artery Disease (CAD) and Peripheral Vascular Disease (PVD)]; Cerebrovascular diseases (such as Transient Ischemic Attack (TIA) and stroke); as well as heart diseases [i.e., Myocardial Infarction (MI), *angina pectoris*, and Heart Failure (HF)]. CVD is the number one killer globally, being at fault for ∼17.9 million deaths globally in 2019, which equates to 32% of all deaths globally (World Health Organisation, 2021).

Atherosclerosis is the leading cause and underlying condition of the vast majority of CVDs. Atherosclerosis *per se* is a complex, multi-factorial, progressive, chronic disease, caused by dyslipidaemia, and low-grade inflammation (Garg et al., 2015) (Poznyak et al., 2020). It is characterised by a slow, but persistent, accumulation of lipids, and cell debris, forming fibro-fatty lesions, overtime developing into an atheromatous plaque, which in turn narrows the lumen of the major large and medium arteries (Low-Wang et al., 2016) (Rafieian-Kopaei et al., 2014). Atherosclerotic cardiovascular disease (ACVD) is also referred to as the ‘silent killer’, due to the sub-clinical, slow-going but sustained progression of the disease throughout the lifetime of an individual, appearing asymptomatic until becoming clinically relevant (or symptomatic), after the occurrence of a CVD event (i.e., MI, TIA, or stroke).

Type-2 Diabetes Mellitus (T2DM) is an ever-growing issue globally and is considered to have reached the status of a global pandemic in the 21st century (Poznyak et al., 2020). Associated CVDs is the primary contributor to mortality amongst diabetes patients (Ginter and Simko, 2012). There is a mutual ‘Casual-Effect’ relationship between ACVD and T2DM: on one side, the establishment of diabetes leads to a number of microvascular complications (such as nephropathy, retinopathy, and neuropathy), as well as contributing to greater macrovascular complications (including atherosclerosis, which may ultimately lead to the forementioned CVDs). On the other side, pre-established ACVD enhances the risk to develop T2DM (Poznyak et al., 2020).

Inflammation is believed to be a driving force in the development of atherosclerosis-associated diabetes complications (ADCC) (Ridker, 1999). The idea that phlogistic events are major contributor to all stages of atherosclerosis was first presented by Sir William Osler in 1907 (Osler and McCrae, 1907). Diabetes accelerates atherosclerosis by amplifying inflammatory processes in the major cell types involved in the pathogenesis of diabetes-associated atherosclerosis (DAA), including the endothelium and the monocyte-macrophage-foam cell axis, thus leading to higher rates of mortality, and morbidity amongst diabetic patients (Yuan et al., 2019).

In atherosclerotic patients, abundant circulating levels of high-sensitivity C-reactive protein (hsCRP) and interleukin-6 (IL-6) are considered biomarkers of inflammation, associated with an increased risk of cardiovascular events, independently of cholesterol levels (Hwang et al., 2017). Furthermore, hsCRP is closely related to the progression of plaque development (Wang et al., 2014), and IL-6 has been identified as independently associated with the risk of major cardiovascular events (such as MI, HF, and mortality), thus reflecting a pathophysiological role in the development of these cardiovascular events (Held et al., 2017). More recently, together with our local collaborators in Mater University Hospital (Dublin, Ireland), we have evidenced the mutual relationship between inflammatory biomarkers levels and cerebrovascular events, by demonstrating, on one hand, the predictive role for IL-6 and hsCRP in the recurrence of coronary events post-stroke (Coveney et al., 2022a), and on the other hand, the role played by acute plaque inflammation in the pathogenesis of cerebral thromboembolism (Coveney et al., 2022b). This is in line with the original Ridker’s hypothesis, from 2016, to identify novel biomarkers of atherosclerosis and targets of athero-protection by moving upstream CRP release, along the inflammasome signalling IL-1/IL-6 axis (Ridker, 2016).

Since the major contributors of AADC have been recognised in dysregulated glycemia and dyslipidaemia, the development of glucose-lowering and lipid-lowering drugs provided a beacon of hope for diabetes care because they not only controlled glucose homeostasis, but also exerted a disease modifying action in diabetic patients, by also tackling lipid levels (Drucker, 2018).

Among the many glucose-lowering drugs available, there is growing interest in Glucagon-Like Peptide 1 Receptor Agonists (GLP-1RA), due to its ability to suppress glucagon production and increase glucose-dependent insulin secretion, thus delaying gastric emptying and subsequently improving patient satiety, resulting in weight loss and reduction in systolic blood pressure (Drucker, 2018). More recently, for example, a glucagon-like peptide-1 (GLP-1) analogue, *Semaglutide*, demonstrated cardiovascular protective benefits, showing reduced relative and absolute risk of Major Adverse Cardiac Events (MACE) (defined as CV death, non-fatal stroke, non-fatal MI) when compared to placebo (Husain et al., 2020). A longer acting GLP-1 agonist, *Liraglutide*, has also been shown to decrease the presence of hsCRP by 20%, *via* decreasing key inflammatory pathways (Savchenko et al., 2019). This body of evidence points towards the positive ramifications that GLP-1 agonists exert on CVD by tackling inflammation *via* an anti-inflammatory response (Dhindsa et al., 2020).

Lipid-lowering drugs, such as statins and Pro-protein Convertase Subtilisin/Kexin type 9 inhibitors (PCSK9i), have been shown to decrease cholesterol levels by 50%, thus reducing the so-called Residual Cholesterol Risk (RCR). Despite a significant reduction in RCR, as demonstrated by Low-Density Lipoprotein Cholesterol (LDL-C) levels, patients remain vulnerable to increased inflammation due to persistent high levels of circulating hsCRP (Aday and Ridker, 2018), and they remain at the greatest risk for MACE (Lin et al., 2017). However, it has been evidenced the ability of statins to also diminish the so called Residual Inflammatory Risk (RIR) *via* attenuation of circulating hsCRP in randomised clinical trials, ultimately leading to a reduction in cardiovascular risk (Pradhan et al., 2018) (Aday and Ridker, 2019).

Resolution of Inflammation (RoI) is not a passive process but indeed a dynamic phenomenon, finely tuned by several soluble mediators, including the so called Specialised Pro-resolving Mediators (SPMs), such as Lipoxins (LXs), Resolvins, Protectins, and Maresins (Kotlyarov and Kotlyarova, 2022). Thanks to the ground-breaking work of Godson and collaborators, our group has been mainly focusing on elucidating the role of LXs in RoI and as a novel therapeutic target. Since their discovery in Stockholm in the 1990s (Clish et al., 1999) (Clish et al., 2000) (Serhan, 2005) LXs have paved the way for a deeper understanding in the spontaneous resolving phases of inflammation, which have been previously characterised into the cessation of inflammatory triggers, followed by a promoted dispersal of inflammation, as reported in ‘Taber’s Cyclopaedic Medical Dictionary’ (Taber, 1970). The anti-inflammatory properties of LXs have been clearly identified over the past two decades. Their action is mainly attributed to a controlled inhibition of neutrophil infiltration and subsequent stimulation of a non-phlogistic recruitment of monocytes to sites of inflammation (Serhan, 2011). The exact mechanism of action of LXs and other SPMs, is still under investigation, and thus far it has been clearly highlighted their key role in the return of a damaged tissue to homeostasis (*catabasis*), especially in the context of diabetes complications (de Gaetano et al., 2018). According to Taber, the acute inflammatory process has been divided into four phases: inflammation initiation, transition, resolution, and return to homeostasis (Taber, 1970) (Serhan et al., 2020). A deeper understanding of RoI has provided the foundations for extensive research into new eras in pharmacology, such as the development of anti-inflammatory and pro-resolving approaches and triggered the activity of chemists to design and develop stable analogues of the endogenous mediators of RoI, including the synthetic LX mimetics (sLXms), with a view to attenuate chronic inflammation by bolstering the defence against persisting low-grade inflammatory processes.

LXs and various generations of LXA_4_ stable analogues (sLXms) [Including benzo-lipoxins (O’Sullivan et al., 2007), pyridines (Duffy et al., 2010), quinoxalines (de Gaetano et al., 2021; Galvão et al., 2021), imidazoles (de Gaetano et al., 2019) and bicyclo-pentanes (Owen et al., 2022)] have proven effective in the attenuation of inflammation and the promotion of RoI in several *in vitro* adipose and/or vascular inflammatory models; As well as numerous *in vivo* murine models of obesity (Börgeson et al., 2015), vascular inflammation, peritonitis (de Gaetano et al., 2019) (de Gaetano et al., 2021), paw swelling (de Gaetano et al., 2021), renal fibrosis (Brennan et al., 2013), diabetes kidney diseases (Brennan et al., 2018a) and arthritis (Galvao et al., 2021). The efficacy and potency of LXs have been also validated in a novel translational atherosclerosis *ex vivo* model (Brennan et al., 2018b). Taken together, these data strongly suggests a potential role for sLXms as novel therapeutic avenues in Resolution Pharmacology).

Despite the global prevalence of atherosclerosis, there still remains a severe lack of diabetes therapeutics that demonstrates the ability to reduce cardiovascular events and/or mortality (Dhindsa et al., 2020). Recent advancements have been made with the use of *colchicine*, with promising results from the Low-Dose Colchicine (LoDoCo)-2 trial (ACTRN12614000093684) showing a significantly reduced occurrence of CVD-related death, MI, or stroke. These results bolster previous findings in earlier trials, such as the LoDoCO trial and the Colchicine Cardiovascular Outcomes Trial (COLCOT), which demonstrated protective properties of colchicine (Nidorf et al., 2020). It has become apparent that RIR and RCR are two elements of atherosclerosis treatment that must be targeted in a combinatorial manner (Aday and Ridker, 2019).

In this review, we are therefore posing the rationale for the use of novel synthetic mimetics of endogenous regulators of inflammation, namely sLXms, as an adjuvant therapeutic to gold-standard therapies, to simultaneously reduce both RIR and RCR, focusing on the role of an impaired RoI in diabetes, as a primary contributor of atherosclerosis development and novel therapeutic target in the Resolution Pharmacology of AADC.

## Pathogenesis of atherosclerosis

**FIGURE 1:**
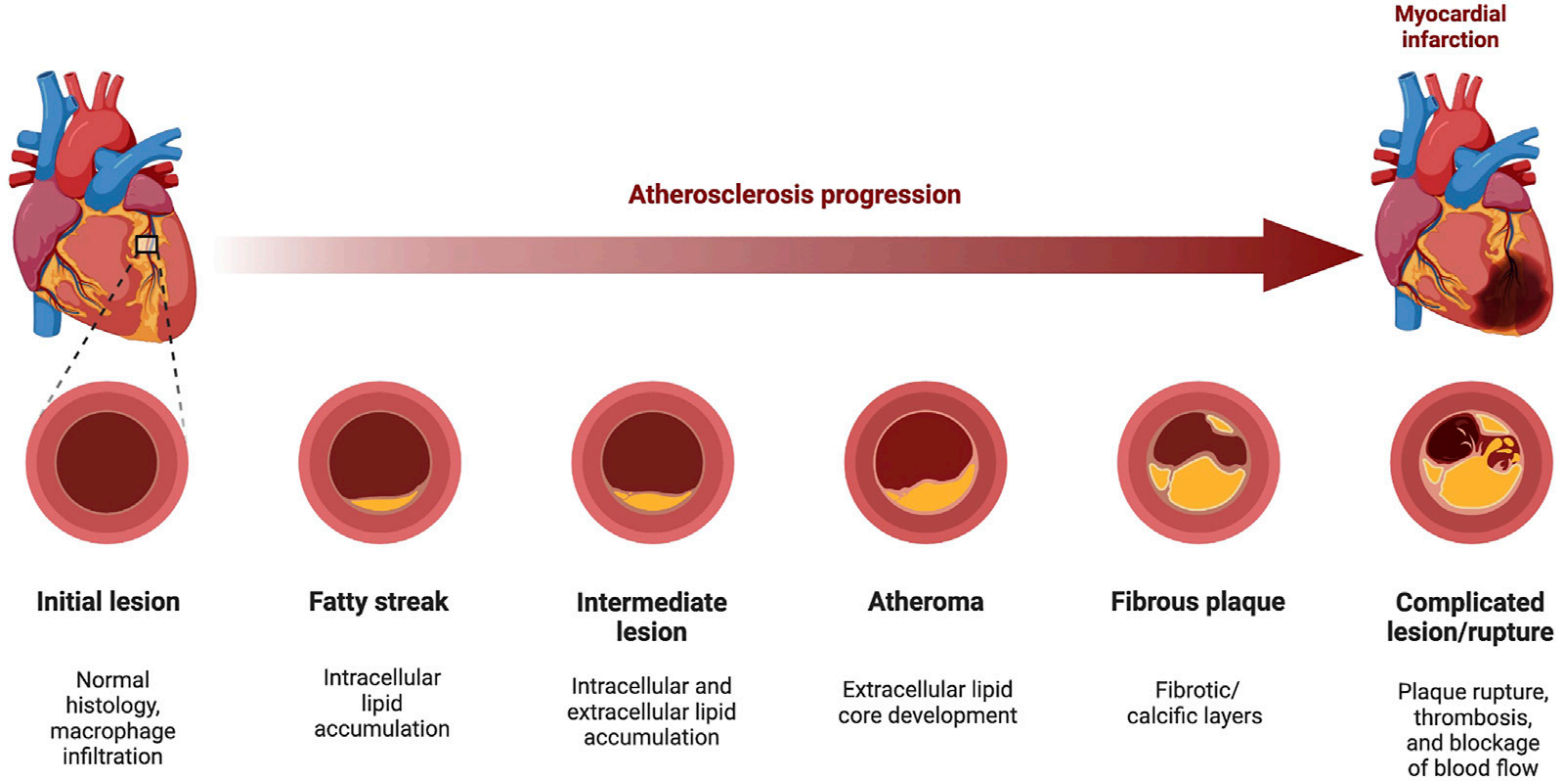
Progression of Atherosclerosis. The development of atherosclerosis is a 5-step process from the initial development of the fatty streak into an atherosclerotic lesion, through to the development/rupturing of fibrofatty atheroma. During atheroma development, patients develop hypertension as the artery becomes narrowed or completely blocked, forcing the heart to pump harder to pass blood through. Elasticity of the vascular tissue allows some flexibility as the lumen narrows, and the mediators of this elasticity also promote the formation of the fibrotic cap on the plaque, providing stability. However, the subsequent rupturing of this plaque can lead to a myocardial infarction by blocking cardiac blood supply or may even cause plaque debris to travel to blood vessels, reaching distant organs (i.e., brain, kidneys, heart) and causing an infarction (heart or kidneys), or a stroke (brain). In this diagram, an example of coronary atherosclerosis development is depicted. Atherosclerosis can also form in the aortic arch, as well as in carotid and femoral arteries (Figure made with BioRender.com).

Atherosclerosis has a very complex etiopathogenesis at both cellular and molecular levels. Figure 1 (Biorender, 2022) below shows the development of an atherosclerotic plaque, from the sub-clinical sign of a fatty streak development to the formation of the atheromata, and the subsequent rupture or erosion of the plaque, which is also associated to thrombus formation (*atherothrombosis*).

A primary step in the initiation of atherosclerosis is an increased lipolysis and lipidaemia, due to the lack of insulin regulation in T2DM (Viola and Soehnlein, 2015). The upregulated lipolysis contributes to an increase in free fatty acids in circulation, which in turn leads to the release of pro-inflammatory cytokines, such as Tumour Necrosis Factor alpha (TNF-*α*) (Sobczak et al., 2019), an increased formation of LDL-C, and a decrease in High-Density Lipoprotein Cholesterol (HDL-C) concentration. The decreased concentration levels of HDL have an intensifying effect on subsequent increase in LDL concentration (Acton et al., 1999). Increased plasma cholesterol levels alter arterial endothelial permeability, allowing circulating LDL present to infiltrate through the more lose endothelial cells junctions, causing a damaged to the endothelial wall. LDL molecules accumulate and adhere to the vascular sub-intima layer *via* cell adhesion molecules (CAMs) (such as VCAM-1, ICAM-1, ECAM-1), selectins (i.e., E-Sel, P-Sel), and proteoglycans (Burke-Gaffney et al., 2002) (Bergheanu et al., 2017) in the extracellular matrix (ECM). Infiltrated LDLs are vulnerable to the action of Reactive Oxygen Species (ROS) for oxidative alteration of their structure into oxidised-LDL (ox-LDL) (Burke-Gaffney et al., 2002). Ox-LDL serves as a potent chemoattractant for monocytes and recruits them to the site of injury (due to inflammatory stress triggers or to endothelial damage), where they are differentiated to macrophages, with a high phagocytic ability, which allow engulfment of trapped ox-LDL particles, thus turning the macrophages into foam cells (Ortega-Gomez et al., 2013) (Bergheanu et al., 2017). Cyclical uptake of ox-LDL and sustained accumulation of foam cells induce endoplasmic reticulum stress, thus leading to the apoptosis of foam cells (Ortega-Gomez et al., 2013).

Circulating monocytes respond to the inflammation and penetrate the damaged tissue, allowing for their differentiation into macrophages. The phenotypic development of the macrophages, however, relies greatly on their extracellular environment: for example, it depends on the presence in the ECM of polarising cytokines (including TNF-*α*, IL-1*β*, IL-10); attracting chemokines (i.e., CXCL4); differentiation-stimulating growth factors [i.e., macrophage- or granulocyte macrophage-colony stimulating factors (M-CSF or GM-CSF)], (Gleissner et al., 2010) (Italiani and Boraschi, 2014) (Orekhov et al., 2019). Primary macrophage phenotypes (such as the pro-inflammatory M1 and the anti-inflammatory M2) are well established and characterised; whilst several sub-phenotypes are still under investigations.

At present, we are able to identify various subtypes of macrophages, with different abilities in terms of phagocytic activity, oxidative capability, scavenging action, iron handling etc: for example, M2-a (wound healing); M2-b (regulatory); M2-c (auto-immunity related); M2-d (cancer-related); M-ox (anti-oxidative), M-heme/Hb (iron handling) (Barrett, 2020).

Such plethora of extreme and intermediate phenotypes is possible due to the action of the microenvironment, switching *on* or *off* different macrophage intracellular signalling pathways, thus conferring a high level of plasticity to this key cellular player. The relative distribution of macrophage subtypes is at the heart of the exacerbation and development of atherosclerosis due to the primary role played by inflammation in disease progression. In fact, macrophages are responsible for the aforementioned cyclical uptake of ox-LDL, *via* the scavenger receptor-A family and the subsequent formation of foam cells (Malekmohammad et al., 2021), the sustained inflammation of the affected area, and the development of the fatty streak and necrotic core, as a result of a defective efferocytosis (Viola and Soehnlein, 2015). It is believed that there is an imbalance in the abundance of the pro-inflammatory and anti-inflammatory macrophage phenotypes, with a significant difference in the M1/M2 ratio in a cohort of people with coronary artery disease and without, tipping the balance in favour of the pro-inflammatory phenotype, which is correlating with disease severity (Hirata et al., 2011).

As the disease progresses, the atheroma grows and steadily narrows the lumen of the blood vessel. Simultaneously, vascular Smooth Muscle cells (vSMC), the main cells forming the subintima layer (*tunica media*), migrate to the intima layer, changing towards a more proliferative phenotype, in order to form a fibrous collagen cap on the plaque, providing stability and preventing interaction between the necrotic core within the plaque, and the external lumen (Singh et al., 2002; Salekeen et al., 2022). Advanced plaque formation is mainly attributed to an inefficient clearance of late apoptotic bodies (i.e., neutrophils) by macrophages, a non-phlogistic process known as *efferocytosis*. This non-inflammatory type of phagocytosis is independent of classical beta-2 integrin-activation.

Effective efferocytosis removes apoptotic bodies without eliciting an inflammatory response, hence preventing a build-up of cell debris, which would ultimately lead to the development of necrotic corpses and subsequent exacerbation of inflammation *via* necrotic core formation. The necrotic area, consisting of isolated lipid pools, irreversibly alters the structure of the lumen. In fact, effective efferocytosis triggers phagocyte re-programming and the macrophages undergo repolarisation, switching their phenotype from a pro-inflammatory phenotype (M1) to an anti-inflammatory phenotype (M2), which is coupled with the release of anti-inflammatory chemokines such as TGF-*β* and IL-10 (Fadok et al., 1998) (Sugimoto et al., 2016). However, the chronic inflammation present in atherosclerosis continues to cause havoc as the pro-inflammatory cytokines released (such as TNF-*α* and IL-1) recruit more macrophages and stimulate the production of growth factors responsible for vSMC production at the inflamed site. Growth factors, such as platelet-derived growth factor (PDGF) and fibroblast growth factor-1 (FGF-1), as pivotal molecules in the formation of a fibrous cap, provide stability to the atheroma and segregate the necrotic core from the blood (Singh et al., 2002).

Impaired efferocytosis (which will be discussed later in more detail) is a tightly regulated process wherein both professional and non-professional phagocytes clear apoptotic cells in order to maintain tissue homeostasis and prevent the development of chronic inflammation after an acute disease (Doran et al., 2019). It has been suggested that the efferocytosis ability of foam cells is altered in atherosclerosis, allowing them to undergo secondary necrosis, hence generating a highly inflamed, necrotic core, which could be central to the development and persistence of atherosclerotic plaque (Burke-Gaffney et al., 2002).

As the inflammation response becomes sustained and unresolved, acute inflammation becomes a chronic process, by which inflammatory cells continue to attack the fibrous cap (providing the plaque stability) and erode it. Key modulators of this erosion are matrix metalloproteinases (MMPs). In atherosclerosis, MMPs play a dual role: in fact, some MMPs (i.e., MMP-12) are beneficial, and others (i.e., MMP-9) appear to be harmful. MMPs generally have a role in the degradation of ECM protein, however, in atherosclerosis, certain MMPs (such as MMP-12) have a protective role *via* maintenance of homeostasis in surrounding tissue, as they have been associated with the regulation of acute inflammatory responses through proteolysis of specific chemokines, disabling their receptor-binding capabilities and thus preventing further neutrophil recruitment (Dean et al., 2008). On the other hand, harmful MMPs are present, such as MMP-9 which has been found to have altered expression in atherosclerosis. This impaired expression in turn promotes ECM degradation, allowing further infiltration of inflammatory cells, and increased migration of vSMCs which consequently increases vascular endothelial growth factor (VEGF) production, promoting neovascularisation of the plaque and increasing plaque instability (Newby, 2007) (Li et al., 2020). Importantly, histopathological studies showed an abundance of MMP-9 in key regions, promoting disease progression and plaque instability, in both the necrotic core and at the fibrous cap (Jiang et al., 2012).

**FIGURE 2:**
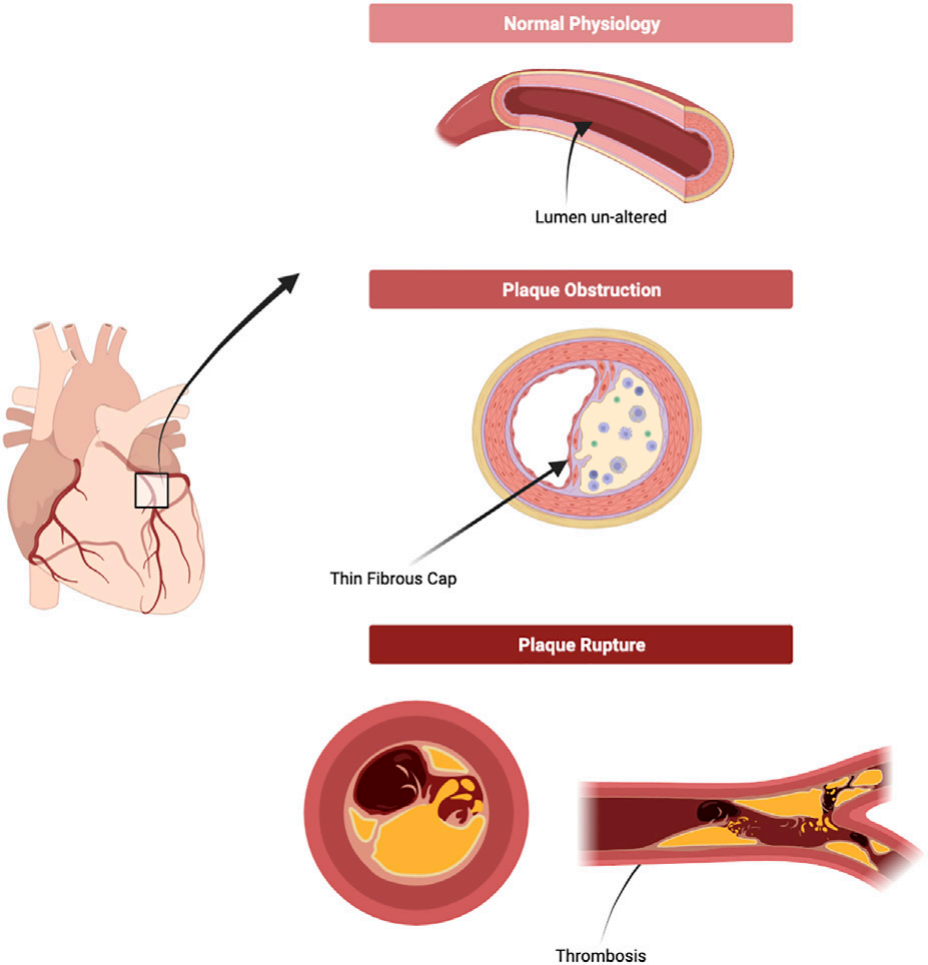
Plaque formation and Rupture. As the plaque develops, the lumen consequentially narrows as the necrotic core of the atheroma alters the structure of the blood vessel irreversibly. A narrower lumen increases blood pressure, putting more strain on the blood vessel and on the plaque. On top of this, inflammatory bodies continue to erode and disrupt the plaque, increasing the likelihood of rupture. Upon rupture, contents of the necrotic core and fragments of the plaque are released freely into the blood vessels and are the leading cause of thrombosis or thrombo-embolism. (Figure made with Biorender.com).

Finally, late stages of atherosclerosis development consist in the rupturing of the plaque (Figure 2) (Biorender, 2022). There are, however, a number of different terms to describe this final phase alongside plaque rupture, such as ‘Plaque fissuring’, ‘Intraplaque haemorrhaging and erosion’, though they are often used interchangeably (Lutgens et al., 2003). ‘Plaque rupture’ was defined as “an area of fibrous cap disruption whereby the overlying thrombus is in continuity with the lipid core” by Virmani et al. in Virmani et al. (2000), and intraplaque haemorrhage was defined as “deposition of blood products inside the plaque and is not necessarily associated with atherosclerotic plaque rupture” by Stary in (Stary, 2000). Nonetheless, this rupturing of a plaque is the primary cause of thrombosis in patients and has contributed to the increasing number of MI, TIAs, and stroke globally (Bentzon et al., 2014).

## Novel targets in the resolution of atherosclerosis

### Inflammatory Response and its dysregulation

There is an absence or reduced expression of a resolution phase wherein a switch from pro-inflammatory to anti-inflammatory/pro-resolving cell recruitment occurs, allowing for appropriate clearance of these pro-inflammatory cells, macrophage reprogramming to an anti-inflammatory and regenerative phenotype, as well as tissue healing and regeneration. Our focus in this review is to highlight the role of LXs in inflammation resolution. LXs are endogenously generated eicosanoids belonging to a group of SPMs generated from omega-6 polyunsaturated fats, which are considered to play a vital role in the resolution of inflammation. The ROS molecules present in the inflammatory *milieu* elicit *in vivo* and *in vitro* lipid peroxidation, wherein there is oxidative decomposition of omega-3 and -6 polyunsaturated fats of phospholipids for LX production. Research suggests that arachidonic acid is a vital LDL present as an endogenous phospholipid in biological membranes or through dietary supply of linoleic acid or animal protein (Kotlyarov and Kotlyarova, 2022). With the resolution of inflammation being a driving factor in atherosclerosis prevalence and development, there is growing consensus into the potential role that synthetic lipoxin mimetics may possess as a future treatment for this disease.

Despite great success witnessed in the use of the aforementioned lipid and glucose-lowering drugs, the role of inflammation in the progression of atherosclerosis cannot be understated or underestimated. Research into the underlying mechanisms surrounding disease progression has revealed new targets for tackling inflammation *via* a greater understanding of the mechanistic pathogenesis of this debilitating disease. Genetic and epidemiological studies identified the inflammatory *common soil* between atherosclerosis and other chronic diseases, posing the rationale to introduce an anti-inflammatory approach for these chronic prevalent inflammatory diseases, such as TNF-*α* blockade, IL-1*β* receptor antagonism, and leukotriene blockade, into atherosclerosis therapy (Klingenberg and Hansson, 2009).

Balancing circulating pro- and anti-inflammatory mediator levels is key for driving RoI, as there exists a serious imbalance in the ratio between SPMs and pro-inflammatory cytokines in atherosclerotic lesions, and the scales need to be tipped in favour of these SPMs to resolve inflammation and promote regression of atherosclerosis (Bäck et al., 2019). The concept of athero-regression, firstly hypothesised by Ed Fisher in the early 2000s (Williams et al., 2008) involves more than simply reversing the effects of atherosclerosis due to the many contributing factors, and hence requires broad changes in plaque macrophage transcriptomes to reduce adhesion, promote mobility, and suppress inflammation (Feig, 2014). A number of therapeutic targets have been identified to address this, such as anti-IL-1β, IL-10 promotion, and micro-RNA inhibition.

The IL-1 family are heavily involved in the innate and adaptive immune systems in inflammation. Two isoforms exist, IL-1 and IL-1β, and both have distinct functional profiles by acting at short distances with cell-cell contact, and at long distances, respectively (Libby, 2017). Within the family there is also the IL-1 receptor antagonist (IL-1RA), which serves as a crucial mediator in the balance of the two isoforms (Libby, 2017). Pro-IL-1β is produced by monocytes, macrophages, and dendritic cells to form the active IL-1β after proteolytic cleavage by caspase-1, which then itself is activated by supramolecular assembly to form the NLRP3 inflammasome. NLRP3 inflammasome is responsible for intracellular activation of IL-1β as a result of oxidative stress, but IL-1β can also be activated extracellularly by Toll-like Receptor (TLR) signalling and Damage-associated Molecular Patterns (DAMPs) using cholesterol crystals or oxLDL as signals (Libby, 2017) (Grebe et al., 2018). Early evidence into the efficacy of the therapeutic targeting IL-1β showed promise with reduced lesion development with an IL-1β deficiency, reduced intimal thickening with reduced IL-1 receptor expression, and reduced fatty lesion formation and MI in mice with over-expressed IL-1RA (Ridker, 2019). The development and repurpose from rheumatoid arthritis to CVD of Canakinumab, an anti-IL-1β monoclonal antibody, showed promising results by reducing diet-induced lesion formation and promotion of endothelial cell growth in mice. This encouraging data, along with the pro-coagulant nature and phagocyte recruitment capabilities of IL-1β, prompted the Canakinumab Anti-inflammatory Thrombosis Outcome Study (CANTOS) to determine the role of IL-1β-induced inflammation in atherosclerosis (Aday and Ridker, 2018). The trial showed significant reduction in MACE (15%), and a reduction of 26% in MI, stroke, and CV death. These outcomes were achieved independently of lipid-lowering, which proved that anti-inflammatory therapy had therapeutic benefits in the treatment of atherosclerosis (Aday and Ridker, 2018).

IL-10 promotion is another an anti-inflammatory approach, alternative to the IL-1β targeting. This anti-inflammatory cytokine has proven athero-protective benefits by limiting the immune response and thus protecting the host from harm (Kamaly et al., 2016). This action is mediated by suppression of the signalling pathway associated with IL-1β activation NF-κB, attenuation of foam cell formation, enhancement of efferocytosis, as well as MMP-9 inhibition to protect ECM integrity (Bhattacharya et al., 2022). Studies investigating the protective role of IL-10 showed a 3-fold increase in atherosclerotic lesions in IL-10 deficient mice, alongside significantly higher HDL-C levels in comparison to normal mice, underpinning the protective role of IL-10 (Mallat et al., 1999). Other studies have explained the protective role of IL-10 through the promotion of Suppressor of Cytokine Signalling 3 (SOCS3). SOCS3 inhibits macrophage-mediated Th1 activation and the subsequent release of pro-inflammatory cytokines IL-1, IL-6, TNF-*α*, and MMPs (Carow and Rottenberg, 2014).

micro-RNA are key post-transcriptional regulators of gene expression, some of which may be athero-protective or athero-promoting. miR-33 is an athero-promoting micro-RNA that is involved with a number of genes relating to nutrient sensing, and lipid and energy homeostasis (Afonso et al., 2021). Research shows the involvement of miR-33 in cholesterol excretion and mobilisation (Rayner et al., 2010), FFA oxidation (Dávalos et al., 2011), gluconeogenesis (Ramírez et al., 2013), and autophagy (Ouimet et al., 2017). The athero-protective elements have been examined and it was found that miR-33-induced suppression of the *ABCA1* gene (associated with cholesterol excretion and mobility) reduces circulating HDL-C levels by dampening the expression of ABCA1 transporter (Rayner et al., 2010). Anti-miR-33 therapy yielded a 25%–30% regression in aortic plaque burden in mice by promoting reverse cholesterol transport, increasing HDL-C levels, and promoting cholesterol efflux from macrophages (Afonso et al., 2021). The pathophysiology of atherosclerosis involves a multitude of factors such as pro- and anti-inflammatory cytokines and other cellular mediators, which opens up an entire avenue of potential therapeutic targets for micro-RNA.

### Apoptosis and impaired efferocytosis

As mentioned earlier in this review, the idea of an impaired or defective efferocytosis of apoptotic bodies has been considered a causative factor in the development of a necrotic core in atherosclerosis. Programmed cell death is a controlled mechanism of cell death to maintain cellular and physiological homeostasis by recycling old cells (Yurdagul Jr, 2021). Apoptotic bodies release ‘find-me’ signals into the environment, including phospholipids and nucleotides, to attract macrophages to their location, and they display ‘eat-me’ signals on their cell surface to discriminate between healthy and apoptotic cells, and allow macrophage binding and subsequent engulfment (Ravichandran, 2011).

Efficient removal of these apoptotic bodies is termed ‘efferocytosis’ and is responsible for preventing secondary necrosis of these apoptotic bodies, terminating inflammatory responses, promoting self-tolerance, and activating pro-resolving pathways (Doran et al., 2019). Under normal physiological conditions, apoptotic cells are recognised for clearance in minutes, however, in atherosclerosis, the number of free apoptotic bodies (not targeted for clearance) is significantly higher within an atheroma and a growing lesion, meaning that these bodies are not being rapidly cleared and it has been estimated that the efferocytosis here is reduced by approximately 20-fold compared to normal physiological states (Schrijvers et al., 2005) (Kojima et al., 2017).

Efferocytosis is a tightly regulated mechanism of cell clearing, using a number of soluble mediators for the detection and differentiation of apoptotic cells and healthy cells. These externalised ‘find-me’ signals, such as CX_3_CL1, specific nucleotides (ATP and UTP), and lipids (sphingosine 1-phosphate and lysophosphatidylcholine), bind macrophages upon arrival using surface receptor proteins such as stabilin1/2, T-cell immunoglobulin mucin receptor 1/3 (TIM1/3), and ADGRB1 (Yurdagul Jr, 2021). This complexes, such as the binding of an apoptotic body to TIM1 and stabilin 2, promotes anti-inflammatory and pro-resolving pathways by hindering NF-κB signalling, which subsequently inhibits TNF-*α*, IL-6, and CCL5 and promotes the production of TGF-*β* (Yurdagul Jr, 2021).

### FPR2 signalling

A key area of interest is the promotion of a pro-resolving environment. As previously mentioned, SPMs are mediators of inflammation resolution. They signal through Formyl Peptide Receptors (FPRs), a diverse family of G-protein Coupled Receptors (GPCRs). These FPRs are recognised as Pathogen Recognition Receptors (PRRs) and detect both Pathogen Associated and Damage Associated Molecular Patterns (PAMPs and DAMPs). FPR2 is of particular therapeutic relevance due to its diverse range of ligands. FPR2 signalling provides an interesting complex dynamic to the study of chronic inflammation and its resolution as it has a dual function of promoting inflammation, but also promoting release of anti-inflammatory mediators, depending on the nature of the ligand (Tylek et al., 2021).

FPR2 is considered a master regulator of RoI, inducing an agonist-dependent switch from a pro-inflammatory response to the secretion of pro-resolving mediators, which in turn encourage macrophage switching, towards an M2 phenotype, thus promoting their efferocytic capabilities (Perretti and Godson, 2020) (Tylek et al., 2021).

**FIGURE 3:**
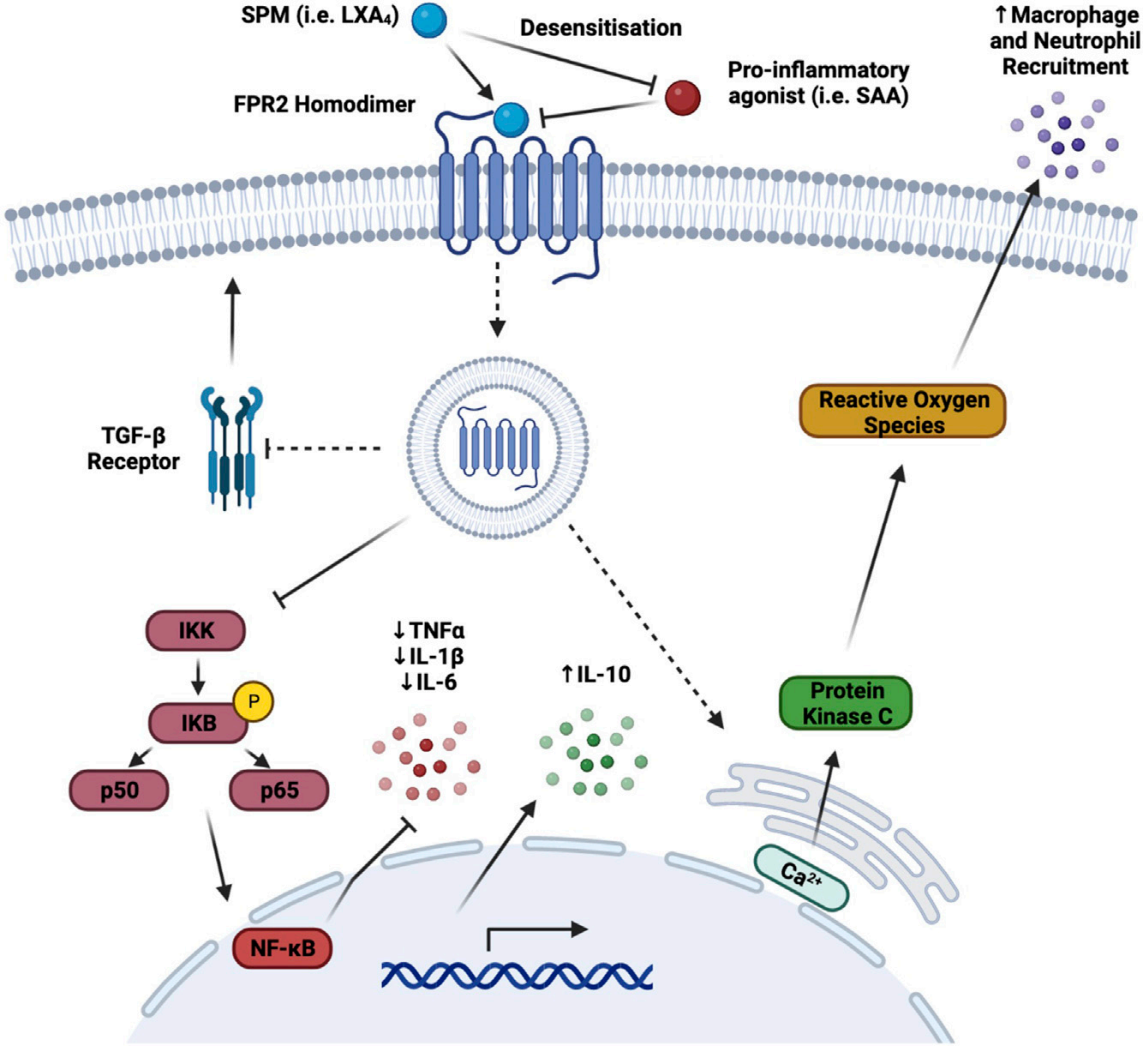
FPR2 Signalling Pathways. The binding of the FPR2 receptor agonist, LXA_4_, has a number of different effects which promote an anti-inflammatory or pro-resolving chain of events. As a receptor agonist, binding of LXA_4_ to FPR2 prevents the binding of other pro-inflammatory mediators to the receptor via pro-inflammatory agonist desensitisation and subsequent internalisation. Downstream of this internalisation, NF-κB is inhibited and as a result, pro-inflammatory cytokine transcription and translation are reduced, thus attenuating their release. Furthermore, internal calcium free ions are released primarily through the endoplasmic reticulum stores. This increased intracellular calcium flux promotes the generation of ROS through protein kinase C activation. The production of ROS increases the conversion of circulating LDL to ox-LDL and thus augmenting macrophage and neutrophil attraction to the site of inflammation, which in turn improves phagocytosis and efferocytosis. Finally, the TGF-β pathway is inhibited *via* a reduction in the expression of its receptor, TGF-βR, which plays a number of key roles, both athero-protective, and atherogenic, modulating the stiffening of arteries and blood vessels in later stages of atherosclerosis. (Broken arrows/inhibitors indicate a downstream effect, whereas a solid arrow/inhibitor indicates a direct effect) (Figure made with Biorender.com).

FPR2 is the receptor for which LXs display the highest level of binding-affinity (Fiore et al., 1994), and its anti-inflammatory effects have previously been discussed and characterised, as seen in Table 1. LXA_4_ is an FPR2 receptor agonist, and its binding prevents the interaction of the receptor with other pro-inflammatory agonists [such as acute phase proteins amyloid *β*, or serum amyloid A (SAA)] (Bena et al., 2012), provoking a subsequent internalisation of the receptor, which causes a desensitisation of FPR2 to the pro-inflammatory action of other ligands (Figure 3) (Biorender, 2022). As a consequence, anti-inflammatory/pro-resolving pathways are activated, whilst pro-inflammatory pathways activation is prevented (Dufton and Perretti, 2010).

**TABLE 1:** Key Publications Discussing the Anti-inflammatory Properties and Functions of sLXms and the FPR2 Receptor.

Article title	Authors	Key feature
Lipoxins, Aspirin-Triggered 15-epi-Lipoxin Stable Analogs and Their Receptors in Anti-Inflammation: A Window for Therapeutic Opportunity	Serhan et al., 2000	Aspirin-triggered 15-epi-Lipoxin
ATLa, an Aspirin-Triggered Lipoxin A_4_ Synthetic Analog, Prevents the Inflammatory and Fibrotic Effects of Bleomycin-Induced Pulmonary Fibrosis	Martins et al. 2009	Aspirin-triggered synthetic Lx analogue
Lipoxin A_4_ and aspirin-triggered 15-epi-lipoxin A_4_ inhibit peroxynitrite formation, NF-κB and AP-1 activation, and IL-8 gene expression in human leukocytes	József et al., 2002	Aspirin-triggered 15-epi-Lipoxin
Novel 3-Oxa Lipoxin A4 Analogues with Enhanced Chemical and Metabolic Stability Have Anti-inflammatory Activity *in Vivo*	Guilford et al., 2004	3-Oxa Lipoxin A_4_ Analogues
Aromatic Lipoxin A4 and Lipoxin B4 Analogues Display Potent Biological Activities	O’Sullivan et al., 2007	LxA_4_ Analogues (1 R)-3a and (1 S)-3a
Synthesis and Biological Evaluation of Bicyclo [1.1.1]pentane-Containing Aromatic Lipoxin A_4_ Analogues	Owen et al., 2022	Bicyclo [1.1.1]pentane-Containing Aromatic Lipoxin A4 Analogues
Annexin A1 Interaction with the FPR2/ALX Receptor	Bena et al., 2012	Annexin-A1
Anti-inflammatory role of the murine formyl-peptide receptor 2: Ligand-specific effects leukocyte responses and experimental inflammation	Dufton et al., 2010	LXA_4_, Compound 43 and SAA

The primary anti-inflammatory action triggered by LXA4 is *via* inhibition of NF-κB transcription factor activation, thus attenuating transcription of genes encoding for inflammatory cytokines and chemokines (de Gaetano et al., 2019). Another anti-inflammatory effect is the inhibition of the TGF-*β* pathway, *via* reduction of the expression of its cognate receptor on the cell surface. Controversially, TGF-*β* is considered to display both anti- and pro-atherogenic properties, since regulating on one hand apoptosis and tissue repair, but on the other also controlling the stiffening of arteries at later stages of atherosclerosis (Toma and McCaffery, 2011).

Pro-resolving actions are also exerted through the release of calcium ions from the endoplasmic reticulum activates protein kinase C, a key component of NADPH oxidase ROS production and ox-LDL generation, which in turn recruit macrophages and neutrophils to the site of inflammation, thus switching *on*, in response, the efferocytic abilities of M2-resolving macrophages (Dorward et al., 2015).

## Current treatments

Currently, there is a lack of treatments to reverse established atherosclerotic CVD (Dhindsa et al., 2020). A number of therapies are currently available on the market for the treatment of some of the instigators or co-morbidities of atherosclerosis by counteracting key metabolic components. These are generally classified into lipid-lowering and glucose-lowering drugs such that they improve dyslipidaemia and regulate glucose levels, thus also modulating insulin release and signalling. The availability of these drugs has provided an intermediate treatment for the time being, while research continues to find this niche treatment.

### Lipid-lowering drugs

The prominent role of dyslipidaemia in the pathogenesis of atherosclerosis highlights lipid metabolism pathways as primary therapeutic targets in the battle against the onset and progression of this disease.

Current management of diabetes-associated atherosclerosis involves intense pharmacological interventions, dietary/lifestyle modifications, or even metabolic surgery, in more extreme cases. The field of research surrounding lipid-led therapeutics has flourished over the last number of years, as there are a number of these in trial at the moment or recently approved (Hegele and Tsimikas, 2019).

Statins represent the first in line treatment for lipid lowering in atherosclerosis and are the most prescribed anti-dyslipidaemic drug due to their efficacy. In the United States, more than 25% of people over the age of 40 are under statin therapy (Marcum et al., 2019). Although initially developed for the treatment of familial hypercholesterolaemia (Nordestgaard et al., 2013), statins exceeded expectations at reducing LDL-C levels by preventing cholesterol production the liver, *via* inhibition of HMGCoA (3-hydroxy-3-methylglutaryl coenzyme A) reductase activity. This prevents conversion of HMGCoA to mevalonic acid and in turn actually increases microsomal HMG-CoA reductase, and increased LDL-C surface receptors to aid in the reduction of circulating LDL-C (Ward et al., 2019). Studies investigating the efficacy of statins found that their use at the highest tolerated dose yielded a decrease of approximately 50% in LDL-C levels (Grundy et al., 2021), while other trials revealed a correlation between statin dosage and increased mortality rates (Rodriguez et al., 2017). Statin intolerance can be an issue for some individual’s, including those at high risk for cardiovascular events. These vulnerable individuals may also not achieve an adequate response from statin therapy due to their elevated risk of CVD, and hence an add-on or alternative therapy may be suggested (Handelsman and Lepor, 2018).

Proprotein convertase subtilisin/kexin type 9 inhibitor (PCSK9i) is a relatively new therapeutic which shows incredible efficacy in trials. It is a key regulator of circulating LDL-C *via* the degradation of the LDL receptor (LDLR). Normal recycling of the LDLR involves binding to LDL, followed by the LDL/LDLR complex being internalised and recycled back to the surface, sometimes 150 times a day (Brown, Anderson, and Goldstein, 1983). PCSK9 then binds to the LDLR and prevents recycling of the receptor by intracellular degradation in a lysosome, thus reducing LDLR present on the cell surface and reducing the amount of LDL-C internalised, increasing LDL-C levels in circulation (Handelsman and Lepor, 2018), thus providing a role for PCSK9i in treatment of atherosclerosis.

Ezetimibe is a selective cholesterol-absorption inhibitor for the treatment of hypercholesterolaemia and hyperlipidaemia by lowering total cholesterol levels, including LDL, Apo-B, and non-HDL particles in patients (Sizar et al., 2022). Ezetimibe differs from other lipid-lowering drugs in that it does not directly affect or inhibit cholesterol synthesis, but rather targets the Niemann-Pick C1-Like 1 (NPC1L1) protein, a sterol transporter responsible for mediating cholesterol ingestion and counter-balancing hepatobiliary cholesterol secretion (Tie et al., 2015). By doing so, it inhibits cholesterol absorption from diet by NPC1L1 and also promotes the production of endogenous cholesterol synthesis, thus reducing LDL, increasing circulating HDL, as well as cholesterol clearance from the blood (Tie et al., 2015) (Sizar et al., 2022). This drug can be used alone or in combination with statins, since it has been shown to reduce LDL levels by 13%–20% (similarly to statins) (Grundy et al., 2019), and significantly reduces hsCRP levels in combination with statins (Arabi et al., 2022), which also attenuate RIR, as previously mentioned (Aday and Ridker, 2019).

### Glucose-lowering drugs

Glucose-lowering drugs have been found to have a dual action in reducing inflammation and CVD, despite their primary function of controlling glucose levels in T2DM. These drugs exert a disease-altering effect on CVDs by modifying certain risk factors (Kim et al., 2019).

Sodium-glucose cotransporter 2 inhibitors (SGLT2i) are a successful form of treatment for the control of blood glucose levels that also contribute to the regulation of insulin and lipid profiles, however, they have also demonstrated positive cardiovascular benefits in some recent studies (Xu et al., 2021). The SGLTi works by preventing the reabsorption of glucose in the proximal convoluted tubule in the kidneys and consequentially promoting the excretion of excess glucose (Kalra, 2014). On average, a non-diabetic human nephron filters 180 g of glucose through renal glomeruli in order to maintain glucose homeostasis, and the reabsorption channels are the passive glucose transporters (GLUTs), whilst the active co-transporters are SGLT1 and SGLT2. The therapeutic benefit of an SGLT2i is that with increased glucose excretion, blood glucose levels can return to homeostasis, which has an indirect cardiovascular benefit, due to the role played by hyperglycaemia in atherosclerosis (Kalra, 2014) (Pahud de Mortanges et al., 2021). Glucose regulation also has an affect on dyslipidaemia, another major player in the development and exacerbation of atherosclerosis. Glucose regulation leads to a catabatic lipolysis, which in turn reduces the inflammatory burden on the cardiovascular system, returns endothelial cells to normal physiology, reduces ROS production, and inhibits foam cell formation (Pahud de Mortanges et al., 2021). With respect to therapeutic benefits in atherosclerosis, SGLTis have shown to improve cardiovascular mortality in MACE and reduce the risk of HF in a meta-analysis by Zhu et al. (2020), and a reduction in the risk of atherosclerosis-associated MACE in another study by Ghosh-Swaby et al. (2020). Zhu *et al.* performed a meta-analysis on 32 trials with more than 55,000 participants and identified a 13% reduction in risk of MACE in patients with T2DM, pre-diabetes, or highly at risk of diabetes with the use of SGLTi. Similarly, they identified a 21% risk reduction in cardiovascular mortality in SGLTi users when compared to controls, and a 32% reduction in heart failure in other meta-analyses. Ghosh-Swaby *et al.* identified a 12% atherosclerotic MACE reduction in comparison to standard care or control groups.

Glucagon-like Peptide-1 receptor agonists (GLP-1RA) are another primary treatment for glucose homeostasis. GLP-1 is a known as an incretin, a peptide hormone released by L cells in the distal ileum and the colon, which functions to release insulin from the *β* cells in the Islets of Langerhans upon nutrient ingestion (Shaefer et al., 2015). The incretin response is responsible for the majority of glucose-dependent secreted insulin from oral glucose load however, GLP-1 has further glucoregulatory responsibilities such that it regulates satiety and delays gastric emptying (Baggio and Drucker, 2007). In T2DM, the incretin effect is diminished and there is a subsequent insensitivity and resistance to the effects of GLP-1. The development of GLP-1Ra allows for persistent activation of the GLP-1R on cell surface to promote insulin secretion, suppresses the secretion of glucagon, and improves satiety (Kim et al., 2019). The potential and efficacy of GLP-1Ra for therapeutic benefits of cardiovascular disease has been examined extensively for a number of years. A primary trial in determining the safety and efficacy of GLP-1Ra was the Liraglutide Effect and Action in Diabetes (LEADER) trial. This double-blind placebo-controlled trial demonstrated the safety and efficacy of a GLP-1RA, Liraglutide, by Novo Nordisk A/S in 2016, showing a reduction by 13% in risk of MACE, a 22% reduction in patient mortality, and a lower rate of non-fatal stroke and MI (Marso et al., 2016). This success could be attributed to an anti-inflammatory secondary feature of GLP-1RA’s. The anti-inflammatory capabilities of GLP-1RA’s were first described back in 2007 by Viswanathan et al. as “non-metabolic actions” of Exenatide, and further research throughout the years have shown GLP-1RA’s to attenuate circulating hs-CRP levels, regulate and lower systolic blood pressure, and reduce inflammatory markers *in vivo* such as TNF-*α*, IL-1β, and IL-6 (Viswanathan et al., 2007) (Pang et al., 2022). They have also been shown to dysregulate key signalling pathways of inflammation, such as NF-κB, MAPK, PKA/STAT3, and PI3K/Akt (Chen et al., 2022). Similarly, dipeptidyl peptidase-4 inhibitors (DPP-4i) is another glucose-lowering drug that has secondary anti-inflammatory effects, and functions to prevent inactivation of circulating GLP-1 (Omar and Ahren, 2014). Studies on their anti-inflammatory capabilities showed a significant reduction in monocyte TNF-*α* expression and a significant increase in monocyte IL-10 expression, promoting a switch to an anti-inflammatory macrophage phenotype (Satoh-Asahara et al., 2013).

### Clinical challenges and unmet needs

Despite the great strides taken in the understanding of mechanisms of action, and the development of effective therapeutics, there are associated negative aspects and gaps that remain in the field. These clinical challenges and unmet needs represent a barrier in the furthering of this already successful field of research. The current gold-standard approach to treatment includes the aforementioned lipid-lowering drugs, as well as surgical revascularization interventions, such as stenting applications (angioplasty) or plaque removal.

The safest and most performed surgical procedure is the removal of the advanced plaque by endarterectomy: Carotid Endarterectomy (CEA) is the elective surgical procedure in preventing cerebrovascular diseases; whilst Femoral Endarterectomy (FEA) is the elective intervention in PVD, due to the intrinsic lower risk of peri-procedural stroke or death, in comparison with other types of intervention (i.e., carotid artery stenting). The selected treatment may depend on whether the patient is symptomatic or asymptomatic, where ‘symptomatic’ is defined as a patient with prior ischemic attack history, but no CVD, and ‘asymptomatic’ is defined as a patient with no prior history of ischemic attacks or CVD (de Gaetano et al., 2016). Several trials have identified a risk reduction in stroke after CEA. This procedure has been recommended to patients presenting with 70%–99% stenosis (percentage of the arterial lumen blocked) in both the EU and the US, after three individual studies (the European Carotid Surgery Trial, the North American Symptomatic Carotid Endarterectomy Trial, and the Symptomatic Veterans Affair Cooperative Study Trial) identified a 16% reduction in absolute risk reduction, and ∼50% relative risk reduction from TIA after 5 years (Bagley and Priest, 2020). Similarly, in asymptomatic carotid stenosis, two other studies (Asymptomatic Carotid Atherosclerosis Study and Asymptomatic Carotid Surgery Trial) on the use of CEA with a smaller percentage of stenosis revealed a statistically significant decrease in absolute risk, and a relative risk of stroke decrease by 4%–6% after 10 years (Naylor, 2017).

It is common knowledge that any surgical procedure poses a risk to health, so a reduction of 4%–6% begs the question of whether an invasive surgery such as CEA is the most suitable approach. A less invasive approach used is Carotid Arterial Stenting (CAS), wherein a stent is inserted into the affected carotid artery to keep the lumen at a desirable diameter for efficient blood supply. A number of studies have been conducted to compare the efficacy of these two approaches, however, the conclusions drawn from these have been contradicting of each other. For example, the rate of restenosis was shown to be increased in CAS in CAVATAS (Carotid and Vertebral Artery Transluminal Angioplasty Study) and CaRESS (Carotid Revascularisation Using Endarterectomy or Stenting Systems), but in EVA-3S (Endarterectomy vs*.* Angioplasty in Patients with Symptomatic Severe Carotid Stenosis) and Mannheim and Karmeli, restenosis was increased in CEA as opposed to CAS (Xin et al., 2019).

With regards to the pharmacological interventions, among lipid lowering drugs, such as statin therapy, despite improving survival to a first cardiovascular event, it has a moderate efficacy in preventing recurrencies (or secondary events). However, there are several associated side effects, including statin intolerance (by which sub-cohorts of patients become non-responders); severe myopathy (promoted apoptosis of vSMCs leading to muscle pain (Gluba-Brzozka et al., 2016)); and an increased risk of diabetes, thus making this latter the most worrying of the mentioned negative aspects of statin therapy. A recent clinical trial (NCT0243084) revealed an increase in insulin resistance of 8% and insulin secretion by 9% compared to the baseline (Abbasi et al., 2021). Despite the beneficial therapeutic effects of statins reducing LDL-C levels, the generalised increase in risk of developing T2DM poses another threat to health, including the development of atherosclerosis due to the aforementioned *mutual* ‘casual-effect’ relationship between ACVD and T2DM.

### Future therapeutic avenues: FPR2 agonists and sLXms

The above discussed limitations of the current (surgical or pharmacological) interventions pose the need for the development of a less invasive approach and/or safer therapeutics. In this context, sLXms provide a beacon of hope for resolving these clinical challenges and unmet needs. The inflammatory nature of atherosclerosis, as well as the *in vivo* instability and costly synthesis of eicosanoids, requires a combined effort of chemists and biologists to design and investigate the protective anti-inflammatory and pro-resolving properties of these endogenously generated mediators. The synthetic mimetics allow for a bioactive and stable compound that will mimic the actions of the endogenous eicosanoid. Serhan and colleagues focused their studies on the development of stable analogues of Resolvins, known as D-series Resolvins (RvDs) (Serhan and Levy, 2018). They found here that RvD1 interacts with DRV1 and Formyl Peptide Receptor 2 (FPR2) for homeostatic and antineutrophil action, respectively, with regards to RoI. With this knowledge, they developed a benzo-RvD1 analogue which proved effective inn early clinical trials for resolving dry eye inflammation (Serhan and Levy, 2018). Other studies have showed success in upregulating the anti-inflammatory cytokine IL-10, and potent action in a number of different areas such as vascular, dermal, obesity, and wound-healing to name a few, and thus it has become a strong candidate for RoI in atherosclerosis (Serhan, 2014). The work from Godson/Guiry, as above described, extensively focused on generating LXA4 mimetics, with a view to be used as adjuvant therapeutics in major chronic inflammatory diseases, including renal and cardiometabolic diseases (de Gaetano, 2023).

LXA_4_ is the most naturally occurring LX which made it a primary target for mimetic development. It elicits a response to inflammation *via* a G Protein-Coupled Receptor (GPCR), namely FPR2. FPRs are GPCRs expressed on the surface of many immune cells, and particular attention has been paid to FPR2 as it is a low affinity and promiscuous receptor capable of recognising a broad number of endogenous and exogenous ligands, such as serum amyloid A or SPMs, to respectively elicit either a pro-inflammatory or an anti-inflammatory response, hence may contribute to the exacerbation of inflammation, or aid in the processes surrounding the resolution of inflammation (Tylek et al., 2021).

FPR2 receptor represents an interesting therapeutic target, since it has been shown to be upregulated in advanced atherosclerotic lesions (Petri et al., 2014) and the use of novel FPR2-agonists, such as synthetic LXA4 mimetics (sLXms), as it will be discussed later in this review, provides an alternative therapeutic approach to achieve RoI.

Endogenous LXs are considered partial non-selective agonists of FPR2, as the maximal receptor activation induced is inferior to the one exerted by exogenous selective full agonist (i.e., W peptide) since they also display intermediate affinity towards other GPCR receptors, such as GPR32, oestrogen receptors, or cysteinyl leukotriene receptors (Tylek et al., 2021). Recent studies showed that also sLXms are partial equipotent FPR2 agonist, when compared to the endogenous ligand LXA4 (de Gaetano et al., 2021), suggesting that they may also be non-selective for FPR2, possibly partially activating to lower extent other GPCRs. sLXms have been proven effective in a series of *in vitro and in vivo* inflammatory models, displaying an IC50 in the picomolar range, showing a very potent action. Among sLXms, imidazole-, oxazole- and quinoxaline-containing sLXms has been investigated in previous studies by de Gaetano et al. (2019) and de Gaetano et al. 2021), successfully identifying two lead candidates, namely, AT-01-KG and AT-02-CT. *In vitro* inhibition of pro-inflammatory pathways by 40%–50% (*via* NF-κB activity attenuation and subsequent pro-inflammatory cytokine release) as well as an enhanced *E. coli* bioparticle phagocytosis by 2.5–3 times, also suggesting the acquisition of an anti-inflammatory/pro-resolving macrophage phenotype *via* FPR2 partial activation (de Gaetano et al., 2019) (de Gaetano et al., 2021). Moreover, carrageenan-induced paw oedema and zymosan-induced peritonitis murine models of acute inflammation also showed a significant neutrophil infiltration at the site of inflammation (de Gaetano et al., 2019) (de Gaetano et al., 2021).

Targeting one of the key regulators of RoI, such as FPR2, provides a unique novel approach to be used in combination with gold standard treatment, which can be potentially administered at a lower dosage than when used alone, thus reducing the risk of unwanted effects of the single treatment and providing a more effective therapy by tackling both RIR and RCR simultaneously.

## Discussion

Atherosclerosis is a chronic, progressive, multifactorial, inflammatory disease which is characterised by a reduction or inhibition in the resolution of inflammation, creating a chronically inflamed environment.

It is a ‘Silent killer’ due to the sub-clinical manifestation and pathogenetic development of the disease until it becomes clinically relevant and detectable. It remains the leading cause of CVD due to the sub-clinical onset and development of the disease. Many advancements have been made in recent years by tackling various components of the disease, with a view of modulating the strong inflammatory and dyslipidaemic components and of identifying the strong genetic contributors (i.e., isolated miRNAs and multi-genetic aetiology).

At present, we still rely on both the surgical and pharmacological interventions, but currently there is still no available treatment capable of tackling the problems of RIR and RCR, simultaneously. Current research shows great promise with the development of novel approaches to achieve RoI, such as the novel therapeutic avenues represented by sLXms. Their have the potential to overcome some of the clinical challenges like the hypothesis of simultaneously reducing the RIR and RCR risks in CV patients; tackling inflammation *via* pro-resolving agents, the identification of novel biomarkers as better and earlier indicators of atherosclerosis, and the tailoring of therapeutic approach by risk stratification (assessing an individual’s needs and optimal treatment options to determine the most effective approach).

The resolution of inflammation is a highly regulated mechanism of action and is not a passive process. The switch from inflammation to a state or resolution involves a gradual shift in the amount of pro-inflammatory molecules and pro-resolving/anti-inflammatory mediators being produced and present at the inflammatory site, which in the case of atherosclerosis coincide with the atheroprone vessel bifurcation regions. The role of SPMs cannot be understated in the context of RoI, as continual research highlights their importance in mediating this switch, especially *via* the FPR2 receptor. These novel master regulators of RoI representing an attractive target for resolution pharmacology is an immense leap forward by providing this new avenue for pharmacological intervention. Their prevalence throughout the body is another benefit as they belong to the largest family of cell surface receptors, GPCRs (Gloriam et al., 2007). They are also considered the most druggable receptor class with reportedly 30%–35% of medicines targeting at least one GPCR (Hauser et al., 2017). Their seven domains with high plasticity contribute to their dual action of promoting inflammation or advocating for its demise, and the many allosteric binding sites they possess benefit the LXA_4_ binding capabilities as the allosteric regions allow for conformational changes to prevent further binding of other ligands and thus promotes the pro-resolving nature of the LX.

The need for pro-resolving therapies is critical since the impaired efferocytosis witnessed in atherosclerosis disease pathology that inevitably leads to the development of a necrotic core within the atheroma, driving chronic inflammation. Following controlled apoptotic death, apoptotic bodies should be cleared without impairment, while enclosed in a double membrane and thus be removed in a non-phlogistic manner. This would prevent a necrotic response to a controlled, cell death in the body, however, in atherosclerosis this mechanism is altered. This efferocytosis impairment is responsible for the chronic inflammation in a number of inflammatory diseases, and there are extremely limited treatments currently available for this, however, the use of miR-33 could be a future therapeutic avenue targeting defective efferocytosis, due to the role of the *abca1* gene in functional efferocytosis (Mehrotra and Ravichandran, 2022).

The importance of the gold-standard therapies at the moment is one that cannot go unnoticed as they have provided a means of intermittent treatment to alleviate the effects of chronic inflammation and slow the disease progression by combatting some of the many factors contributing to atherosclerosis pathogenesis. The 50% reduction in circulating LDL-C provided by statin therapy alone is hugely beneficial in treatment, however, there are still negative effects of statin therapy, as we previously discussed, and hence there is a drive for alternative therapeutics. For this reason, future research cannot be solely focused these next-generation therapeutics such as the sLXms. Additional or alternative therapies also serve to reduce the need for invasive surgeries like CEA, and also provide the opportunity to be used as an alternative therapeutic to the next-generation therapeutics or the gold-standard drugs.

Some investigators have questioned the existence of SPM in sufficient quantities to elicit the reported bioactions and there have been several criticisms of the absence of standardised protocols for detection (O'Donnell et al., 2021; Schebb et al., 2022). However, these assertions have been robustly challenged and re-analysis of data, using revised criteria, has led to outcomes consistent with original interpretations (Dalli, Gonzalez, Serhan 2022). It is noteworthy that, since SPMs typically act as autocrine or paracrine mediators, detection in systemic circulation may be of little relevance to biological activity.

## Conclusion

The purpose of this review is to highlight the current standard of care for all stages of atherosclerosis pathogenesis. Our understanding of disease pathogenesis has changed drastically over the years and more recently, the idea of the need of an effective inflammation resolution and return to homeostasis (*catabasis*) has become abundantly clear. Various cellulate and molecular have been identified, belonging to a variety of different pathways of disease progression, opening up new avenues of research and discovery.

Resolution pharmacology is a front-runner in this field and the therapeutic potential of sLXms through both *in vitro* and *in vivo* assays is extremely promising. sLXms represent the avant-guard of therapeutic strategy(s) with the potential of efficiently reduce both RIR and RCR simultaneously. To this end, the role of sLXms is intriguing, and, in conjunction with gold standard treatment, can ultimately lead to a personalised medicine approach to effectively reach inflammation resolution, thus contributing to the pre-emption (and potentially regression) of many prevalent chronic inflammatory diseases.

## Glossary

**ACVD**: Atherosclerotic cardiovascular disease
**ADCC**: Atherosclerosis-Associated Diabetes Complications
**CAD**: Coronary Artery Disease
**CANTOS**: Canakinumab Anti-inflammatory Thrombosis Outcome Study
**CAS**: Carotid Arterial Stenting
**CE**A: Carotid Endarterectomy
**CVD**: Cardiovascular Disease
**DAA**: Diabetes-Associated Atherosclerosis
FPR: Formyl Peptide Receptor
**GLP-1RA**: Glucagon-Like Peptide one Receptor Agonists
**GLUTs**: Glucose Transporters
**GPCR**: G Protein-Coupled Receptor
**HDL-C**: High-Density Lipoprotein cholesterol
**HF**: Heart Failure
**LDL-C**: Low-Density Lipoprotein cholesterol
**LEADER**: Liraglutide Effect and Action in Diabetes
**LXs**: Lipoxins
**MACE**: Major Adverse Cardiac Events
**MI**: Myocardial Infarction
**MMP**: Matrix Metalloproteinase
**NPC1L1**: Niemann-Pick C1-like 1
**ox-LDL**: Oxidised-LDL
**PAMPs, and DAMPs**: Pathogen Associated and Damage Associated Molecular Patterns
**PCSK9i**: Pro-protein convertase Subtilisin/Kexin type 9 inhibitors
**PRRs**: Pathogen Recognition Receptors
**PVD**: Peripheral Vascular Disease
**RCR**: Residual Cholesterol Risk
**RIR**: Residual Inflammatory Risk
**RoI**: Resolution of Inflammation
**ROS**: Reactive Oxygen Species
**SGLT2i**: Sodium-glucose cotransporter two inhibitor
**sLXms**: Synthetic Lipoxin Mimetics
**SPMs**: Specialised Pro-resolving Mediators
**T2DM**: Type-2, Diabetes Mellitus
**TIA**: Transient Ischemic Attack
**TNF-a**: Tumour Necrosis Factor alpha
**vSMC**: vascular Smooth Muscle Cells
